# Gallic Acid Treats Hypertrophic Scar in Rabbit Ears via the TGF-β/Smad and TRPC3 Signaling Pathways

**DOI:** 10.3390/ph16111514

**Published:** 2023-10-24

**Authors:** Qiannan Li, Chunming Lyu, Daqin Chen, Wanling Cai, Fang Kou, Qiang Li, Hai Wei, Huimin Zhang

**Affiliations:** 1 Department of Dermatology, Shuguang Hospital Affiliated to Shanghai University of Traditional Chinese Medicine, Shanghai 201203, China; liqiannanyes@163.com (Q.L.); 18292921557@163.com (W.C.); 2 Experiment Center for Science and Technology, Shanghai University of Traditional Chinese Medicine, Shanghai 201203, China; chunming83g@126.com; 3 Qinghai Province Key Laboratory of Tibetan Medicine Pharmacology and Safety Evaluation, Northwest Institute of Plateau Biology, Chinese Academy of Sciences, Xining 810008, China; 4 Institute of Interdisciplinary Integrative Medicine Research, Shanghai University of Traditional Chinese Medicine, Shanghai 201203, China; 15236408769@163.com (D.C.); koufang1983@126.com (F.K.); liqiang875@foxmail.com (Q.L.)

**Keywords:** gallic acid (GA), hypertrophic scar (HS), rabbit ear model, TGF-β/Smad, TRPC3

## Abstract

Hypertrophic scars (HSs) develop due to excessive collagen deposition and abnormal fibroblast proliferation during wound healing, significantly impacting patient quality of life. Three dosages of GA ointments were administered to rabbit ear HS models to investigate the potential efficacy and mechanism of gallic acid (GA) on HS. Daily application of ointment was performed on the matrix group, the GA ointment groups, and the silicone gel group for 28 days. (No drug treatment was performed on the skin and model groups as a blank group and vehicle group, and silicone gel ointment was topically administered to the silicone gel group as a positive control group.) Scar specimens were collected for histopathology analysis, RNA sequencing analysis, real-time quantitative polymerase chain reaction, and Western blot analysis at the first, second, and fourth weeks after the treatment. Low-dose and medium-dose GA effectively suppressed HS formation and markedly decreased fibroblast infiltration levels and scar thickness. Moreover, decreased expression of TRPC3 mRNA and TGF-β1, p-Smad2/3, and Smad2/3 protein was observed in the low- and medium-dose GA groups and the silicone gel group. This study provides evidence for the efficacy of GA in treating HS and sheds light on its potential underlying pharmacological mechanisms.

## 1. Introduction

Hypertrophic scar (HS) is a fibrous skin disease resulting from the abnormal proliferation of fibroblasts and excessive collagen deposition. It occurs after skin injuries, especially burns, and can cause severe physical and psychological distress [1,2]. Various medical treatments, including cryotherapy, laser therapy, drug therapy, and compression therapy, are available for HS [3,4,5,6]. However, these treatments may lead to adverse reactions and the appearance of new skin damage [7]. As a form of drug therapy, Chinese herbal medicine treatment has gained attention due to its efficacy, minimal adverse reactions, and economic convenience, providing a novel approach to HS treatment [8]. With increasing research on herbal medicine and its ingredients, numerous studies have shown that some herbal medicine extracts (such as galangin, asiaticoside, and emodin) can treat HS by inhibiting the proliferation of fibroblasts, promoting fibroblast apoptosis, and reducing collagen synthesis [9,10,11].

Gallic acid (GA) is a natural phenolic compound that is widely distributed in various medicinal plants and has diverse biological and pharmacological activities, including antioxidant, anti-inflammatory, antibacterial, anticancer, and antifibrotic properties [12]. Polyphenols, including GA, have demonstrated therapeutic effects in treating chronic skin diseases, such as psoriasis and vitiligo, and promoting wound healing and anti-inflammation [13]. GA could inhibit tumor cell proliferation and promote tumor cell apoptosis without adverse effects on normal cells [14]. Previous studies have demonstrated that GA can induce apoptosis and necrosis in lung and HS fibroblasts [15,16]. However, its effect on HS in rabbit ears has yet to be investigated.

Transforming growth factor beta-1 (TGF-β1), a critical cytokine in fibrotic diseases, plays an essential role in cell growth, differentiation, adhesion, and apoptosis. It also induces excessive extracellular matrix (ECM) deposition [17,18]. TGF-β1 stimulates the differentiation of normal skin fibroblasts into myofibroblasts, which are responsible for a high degree of collagen deposition and contraction, by upregulating the expression of α-smooth muscle actin (α-SMA) [19,20]. Numerous studies have verified that TGF-β1 expression is higher in HS than in normal skin tissue [21]. In the early stages of scarring, fibroblasts synthesize and produce more TGF-β1, VEGF, collagen I, and collagen III, promoting microvessel formation and collagen deposition and leading to tissue proliferation [22]. During HS formation, TGF-β1 binds to and activates its membrane receptor complex (composed of TGF-βRI and TGFβ-RII). Activated TGF-βRI phosphorylates Smad2/3, thereby promoting the transformation of fibroblasts into myofibroblasts, leading to abnormal ECM deposition [23]. TRPC3 (transient receptor potential cation channel subfamily C member 3) is expressed in various tissues and participates in several physiological functions [24,25,26]. In addition, studies have revealed that TRPC3 channels play a crucial role in the pathogenesis of HS. HS contracture is associated with increased TRPC3 expression, inwards calcium flow, and nuclear factor kappa B (NF-κB) activation. Additionally, human keloids show higher TRPC3 expression levels than normal skin [27].

In the present study, three dosages of GA ointment were topically administered to a rabbit ear scar model to investigate the impact of GA ointment on scar tissue, assess the therapeutic potential of GA for HS, and elucidate its underlying mechanisms.

## 2. Results

### 2.1. Effect of GA Treatment on HS

**Figure 1:**
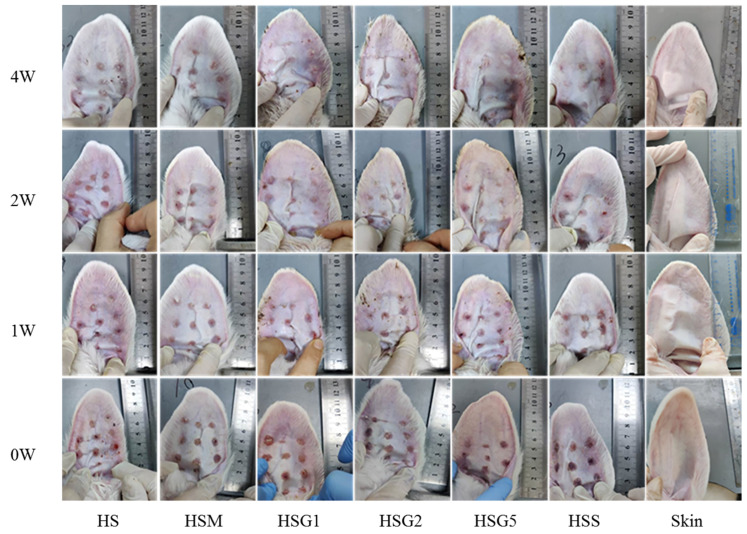
Changes of HS in different groups after the treatments. HS, model group; HSM, matrix group; HSG1, low-dose GA ointment group; HSG2, medium-dose GA ointment group; HSG5, high-dose GA ointment group; HSS, silicone gel group; Skin, normal skin group, *n* = 58.

According to general observations (Figure 1), the wound was epithelialized at the beginning of the treatment. The epithelialized area was confirmed by macroscopic images and a statistical analysis based on the degree of wound contraction. In our study, it was observed that the completion time of wound re-epithelialization after GA and silicone treatment was earlier than that in the HS and HSM groups (Supplementary Figure S5). All groups, except for the skin group, had hard, red, or pink and distinctly raised HSs before drug treatment. After 2–4 weeks of treatment, compared with the model group, the low-dose and medium-dose GA-treated groups and the silicone gel group showed softer scars with reduced thickness and area, and the scars gradually approximated the standard skin color. In contrast, the high-dose GA group showed lightening of the scar color but hardened textures after four weeks of treatment.

### 2.2. Effect of GA on the Histological Characteristics of HS

**Figure 2:**
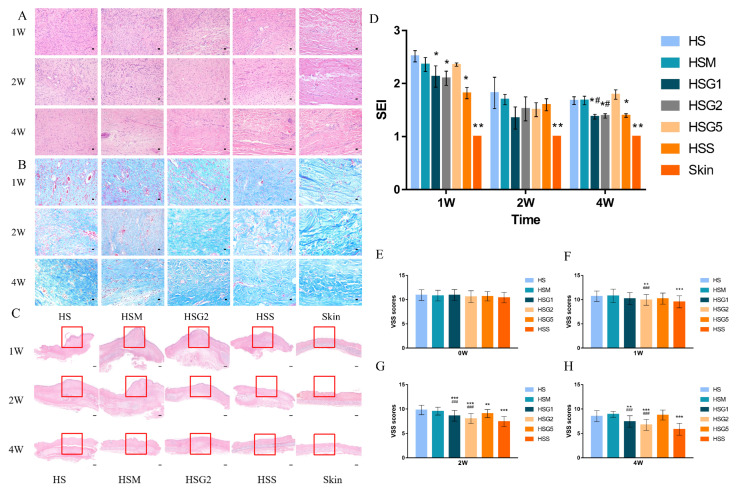
Histological staining, scar elevation index (SEI), and the Vancouver Scar Scale (VSS) scores of each group after the treatments. HS, model group; HSM, matrix group; HSG1, low-dose GA ointment group; HSG2, medium-dose GA ointment group; HSG5, high-dose GA ointment group; HSS, silicone gel group; Skin, normal skin group. (**A**) HE staining of scar tissue after the treatments. Magnification = ×400. Bar = 100 px. (**B**) Masson staining of scar tissue after the treatments. Magnification = ×400. Bar = 100 px. (**C**) HE staining for SEI analysis on rabbit’s ear after the treatments (the marked red boxes in the images were added to label the area on the overview images). Magnification = ×20. Bar = 500 μm. (**D**) SEI in different groups. The SEI was decreased in the HSG1 and the HSG2 groups compared to the HSM group in the scar tissues after 4 weeks. (**E**) VSS scores of each group before the treatments. (**F**) VSS scores of each group after one week of the treatments, *n* = 58. The VSS was significantly decreased between the group of HSG2 and the group of HSM. (**G**) VSS scores of each group after two weeks of the treatments. The VSS scores were significantly decreased in the HSG1 and the HSG2 groups compared to the HSM group, *n* = 48. (**H**) VSS scores of each group after four weeks of the treatments. The VSS scores were significantly decreased in the HSG1 and the HSG2 groups compared to the HSM group. A column represents mean ± SD, *n* = 28. * *p* < 0.05, ** *p* < 0.01, and *** *p* < 0.001, compared with the HS group. ^#^ *p* < 0.05, and ^###^ *p* < 0.001, when the HSG groups were compared with the HSM group. Data were analyzed using one-way ANOVA with Tukey’s post hoc multiple comparisons test.

The HE staining results (Figure 2A and Supplementary Figure S1A) showed that inflammation was attenuated at two and four weeks after treatment with medium-dose GA and silicone gel, respectively. The model group showed denser and thicker collagen fibers, abnormal accumulation of collagen fibers, more content, and disorderly arrangement from Masson staining (Figure 2B and Supplementary Figure S1B). Both the number and density of collagen fibers were reduced, and the disorganization of the collagen fibers’ arrangement was improved in the medium-dose GA and silicone gel groups compared to the model group four weeks after treatment. The immunohistochemical staining for CD31+ blood vessels in different groups is shown in the Supplementary Figure S3. Compared to the HS group, the HSG5 group formed relatively fewer microvessels in 2 weeks after the application of GA, and the same trend was also observed in the HSG1 and HSG2 groups. Moreover, as shown in Supplementary Figure S4, the expression of TGF-β1 was suppressed after the GA treatments on HS.

### 2.3. GA Reduces the SEI Index of HS

To evaluate the effect of GA treatment on the SEI, HE staining was performed, and the results are shown in Figure 2C,D and Supplementary Figure S1C. The SEI index of the low-dose GA, medium-dose GA, and silicone gel groups was significantly reduced after one week of treatment. The SEI index was 2.52 ± 0.11 in the model group, 2.36 ± 0.13 in the matrix group, 2.13 ± 0.20 in the low-dose GA group, 2.10 ± 0.14 in the medium-dose GA group, 2.35 ± 0.03 in the high-dose GA group, and 1.82 ± 0.10 in the silicone gel group. After four weeks of treatment, scarring was considerably attenuated in the low-dose GA group (1.37 ± 0.04), the medium-dose GA group (1.39 ± 0.04), and the silicone gel group (1.39 ± 0.03) compared with the model group (1.67 ± 0.07), suggesting that treatment with GA could inhibit the proliferation of HS and reduce the degree of proliferative augmentation.

### 2.4. Total VSS Score Shows the Effect of GA on HS

Based on the total VSS score (shown in Figure 2E), no significant difference was seen in the total VSS score between the groups before treatment. Notable differences were observed in the medium-dose GA group (10.60 ± 1.22) and silicone gel group (10.42 ± 1.07) compared to the model group (10.93 ± 1.10) after one week of topical application (Figure 2F). There were significant differences among the low-dose GA group, the medium-dose GA group, and the silicone gel group compared with the model group at two weeks (Figure 2G) and four weeks (Figure 2H) after the topical applications, while the high-dose GA group showed a significant decrease in VSS scores compared with the model group at one week and two weeks. Nevertheless, no significant difference or decreasing trend was observed compared with the model group four weeks after topical application. In summary, the color of the scars, the distribution of blood vessels, and the thickness and texture of the scars gradually improved in the low-dose, medium-dose GA, and silicone gel groups.

### 2.5. RNA-Seq Analysis Results

**Figure 3:**
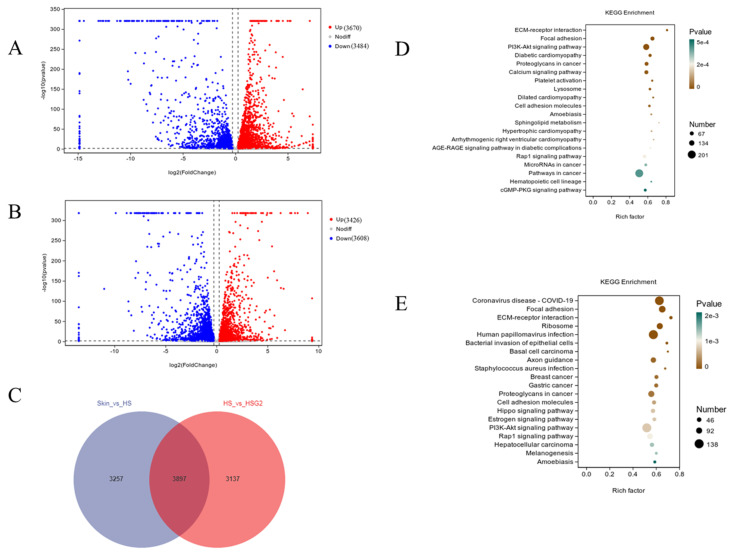
RNA-Seq analysis of overall transcriptomic changes in different groups. (HS, model group; HSG2, medium-dose GA ointment group; Skin, normal skin group.) (**A**) Volcano map of differential gene expression between the Skin group and HS group. (**B**) Volcano map of differential gene expression between HS group and HSG2 group. (Red represents upregulated genes, and blue represents downregulated genes.) (**C**) Venn diagram of the number of shared unique differential genes between groups. (**D**) KEGG enrichment analysis between the Skin group and HS group. (**E**) KEGG enrichment analysis between the HS group and HSG2 group. (The size of the dots in the graph indicates the number of differential genes annotated in the corresponding pathway, and the shade of color indicates the level of significance).

The RNA sequencing analysis revealed that, compared to normal skin tissue, HS tissues had 3670 upregulated genes and 3484 downregulated genes among 7154 total differentially expressed genes (Figure 3A). After the drug treatments, 3426 genes were upregulated and 3608 genes were downregulated, for a total of 7034 differentially expressed genes (Figure 3B). The three groups had 3897 common differentially expressed genes (Figure 3C). The ECM-receptor interaction, PI3K-Akt signaling, and calcium signaling pathways were remarkably enriched in HS tissue compared with normal tissue through the KEGG enrichment analysis (Figure 3D). The heatmap of gene expression showed that TGF-β and Smads played important roles in HS formation (Supplementary Figure S2). A KEGG enrichment analysis of the differentially expressed genes in HS tissue after drug treatments versus those in nontreated scar tissue (Figure 3E) revealed that drug treatments significantly affected ECM-receptor interactions and the PI3K-Akt signaling pathway. These data indicate that scar formation was associated with the accumulation of ECM and the inflammatory response.

### 2.6. Effect of GA on the mRNA Expression of Smad2, Smad3, TGF-β1, and TRPC3 in HS

**Figure 4:**
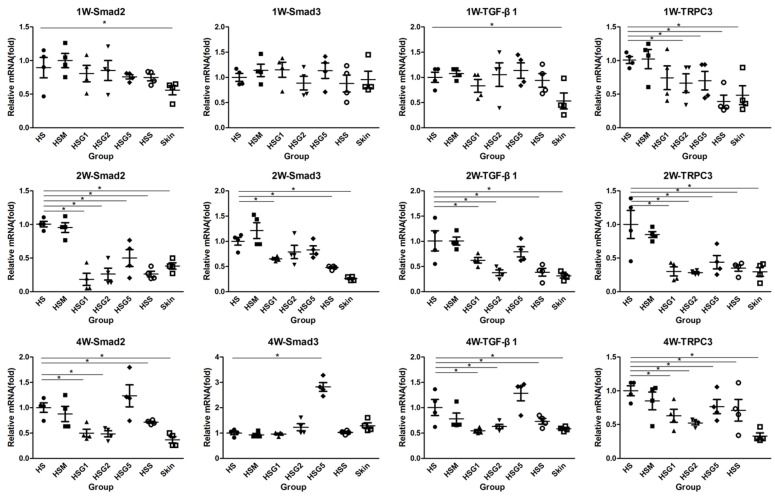
The mRNA expression of Smad2, Smad3, TGF-β1, and TRPC3 in different groups after drug treatment. Data were analyzed using one-way ANOVA with Tukey’s post hoc multiple comparisons test. HS, model group; HSM, matrix group; HSG1, low-dose GA ointment group; HSG2, medium-dose GA ointment group; HSG5, high-dose GA ointment group; HSS, silicone gel group; Skin, normal skin group. A column represents mean ± SD, *n* = 4. * *p* < 0.05, compared with the HS group. The black solid cycle is the sample from HS group, the black solid square is the sample from HSM group, the black solid triangle is the sample from HSG1 group, the black solid inverted triangle is the sample from HSG2 group, the black solid diamond is the sample from HSG5 group, the black hollow cycle is the sample from HSS group, the black hollow square is the sample from Skin group.

RT-qPCR was performed to verify the mRNA expression of Smad2, Smad3, TGF-β1, and TRPC3 in each group at one week, two weeks, and four weeks after treatment (Figure 4). The high-dose GA group showed reduced mRNA expression of Smad2 at two weeks after treatment. After four weeks of treatment, the high-dose GA group failed to reduce the mRNA expression of Smad2. Reduced Smad2 mRNA expression was observed in the low- and medium-dose GA groups and the silicone gel group at two and four weeks of treatment. The mRNA expression of Smad3 was reduced in the low-dose GA group and the silicone gel group after two weeks of treatment, and no significant difference was observed in other groups. In contrast, after four weeks of treatment, Smad3 levels were significantly higher in the high-dose GA group. In addition, the mRNA expression of TGF-β1 was suppressed in the low- and medium-dose GA groups, and the silicone gel group after two and four weeks of treatment. The mRNA expression of TRPC3 was decreased in the medium- and high-dose GA groups and the silicone gel group after treatment.

### 2.7. Effect of GA on p-Smad2/3, Smad2/3 and TGF-β1 Protein Expression in HS

**Figure 5:**
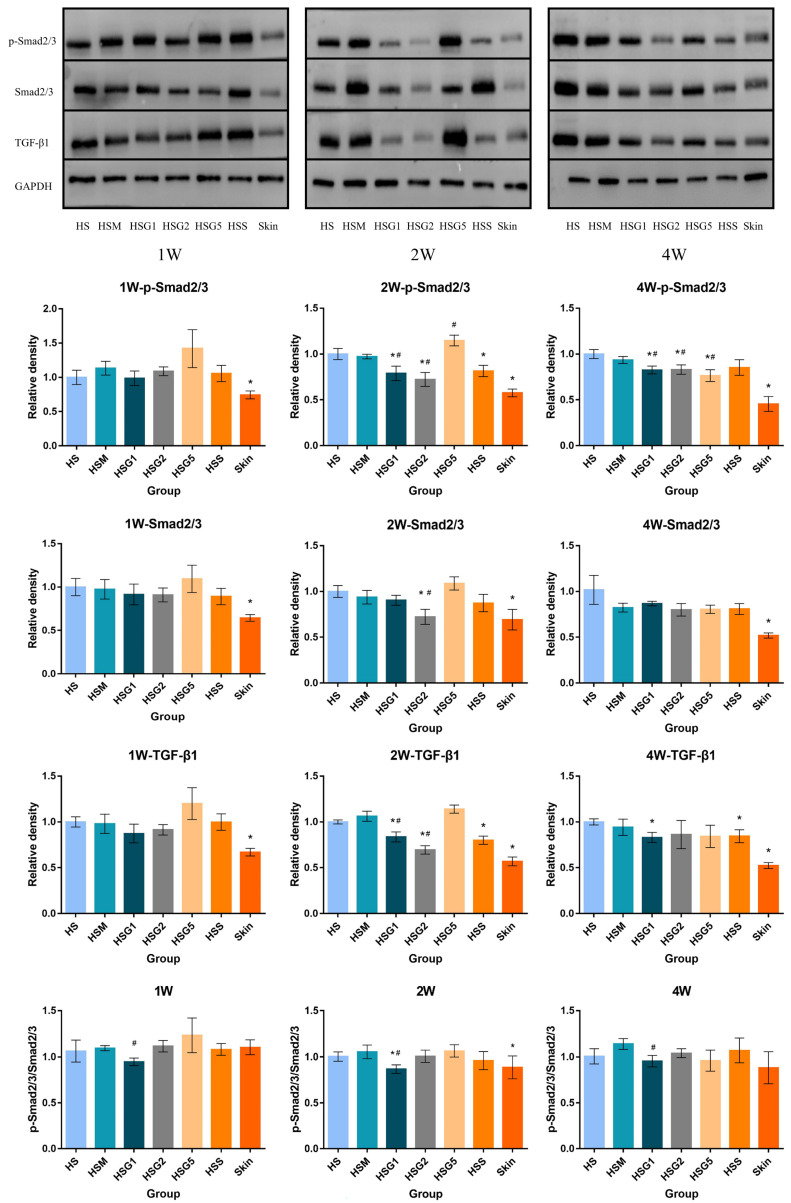
The protein expression Smad2/3, p-Smad2/3, and TGF-β1 in different groups after drug treatment. The activation of Smad was calculated by p-Smad2/3 versus Smad2/3. The Smad activation was significantly inhibited in the HSG1 group in 1, 2, and 4 weeks after GA application. Data were analyzed using one-way ANOVA with Tukey’s post hoc multiple comparisons test. HS, model group; HSM, matrix group; HSG1, low-dose GA ointment group; HSG2, medium-dose GA ointment group; HSG5, high-dose GA ointment group; HSS, silicone gel group; Skin, normal skin group. A column represents mean ± SD, *n* = 4. * *p* < 0.05, compared with the HS group. ^#^ *p* < 0.05, when the HSG groups were compared with the HSM group.

The protein expression levels of p-Smad2/3, Smad2/3, and TGF-β1 were verified by Western blotting in each group at one week, two weeks, and four weeks after treatment (Figure 5). No significant changes in the protein expression of p-Smad2/3, Smad2/3, and TGF-β1 were observed one week after treatment. The protein expression of TGF-β1 and p-Smad2/3 decreased in the low- and medium-dose GA groups and the silicone gel group after two weeks of treatment. Additionally, Smad2/3 protein levels were reduced in the medium-dose GA group. After four weeks of treatment, the low-dose GA group had reduced the TGF-β1 and p-Smad2/3 protein expression. Meanwhile, at four weeks after treatment, p-Smad2/3 protein levels showed a reduction in the low-dose, medium-dose, high-dose, and silicone gel groups. Smad activation was inhibited in the HSG1 group at 1, 2, and 4 weeks after GA treatment (Figure 5).

## 3. Discussion

HS typically develops after surgery, trauma, and burns. Patients experience psychological stress from clinical issues such as pain, itching, and scar contracture [28]. The annual sales of anti-scar medication exceed $12 billion, highlighting the importance of optimizing scar treatments [29,30]. Despite many available therapies for HS, they have yet to meet the demands of patients. The current treatment options remain insufficient, leaving much room for improvement [8]. Therefore, developing new methods for the drug treatment of HS is necessary to address this clinical problem.

The scar model of rabbit ears was used in our study. Masson’s trichrome staining was employed to identify an increase in fibroblasts and microvessels during the early stage of hypertrophic scarring. The distribution, thickness, and density of collagen between H&E and Masson staining were compared. On Day 7 after the operation, there were noticeable increases in H&E and Masson staining in the HSs vs. the adjacent uninjured skin (model group). Compared to those in the skin group (control group), collagen fibers were significantly thicker, denser, and more tangled in the HS group. In contrast, after treatment with GA, a thinner dermal layer, a significant reduction in collagen deposition, and thinner collagen fibers with a relatively more organized pattern were found in HS (Figure 2A,B). On Day 28 after the operation, all groups showed a substantially reduced scar prominence compared to that on Day 7. On Day 28, significant differences in scar thickness and histological structure remained between groups, and the HSG2 group showed finer collagen fibers and reduced density. However, there are some limitations to our study. Masson trichrome staining provides information about the amount of collagen and localization of collagen deposition. Compared to Masson trichrome staining, picrosirius red collagen staining and microscopic evaluation under polarized light could better identify mature and immature collagen and provide more information about the structure of collagen ECM. Therefore, we plan to implement picrosirius red collagen staining in future studies.

Gallic acid (GA) is a natural phenolic compound that is widely distributed in various medicinal plants with diverse biological and pharmacological activities, including antioxidant [31,32], anti-inflammatory [33,34], antibacterial, anticancer [35,36], and antifibrotic properties [37]. Recent studies have shown that GA could be used as a therapeutic agent for keloids. GA reduced the proliferation, migration, and invasion of keloid fibroblasts and induced apoptosis by inhibiting the AKT/ERK signaling pathway [1]. HS and keloids are both skin diseases characterized by pathological scarring and fibrous hyperplasia. However, they differ in the duration and intensity of inflammation [38]. In pathological scarring, the mRNA and protein expression of TGF-β1, PI3K, AKT, and mTOR were significantly higher in fibroblasts than in normal skin tissue [39]. The AKT/ERK signaling pathway could enhance the phosphorylation of Smad2/3, which, in turn, affects the TGF-β1/Smad signaling pathway, and inhibiting the TGF-β1/Smad pathway could be beneficial for treating pathological scarring [40].

**Figure 6:**
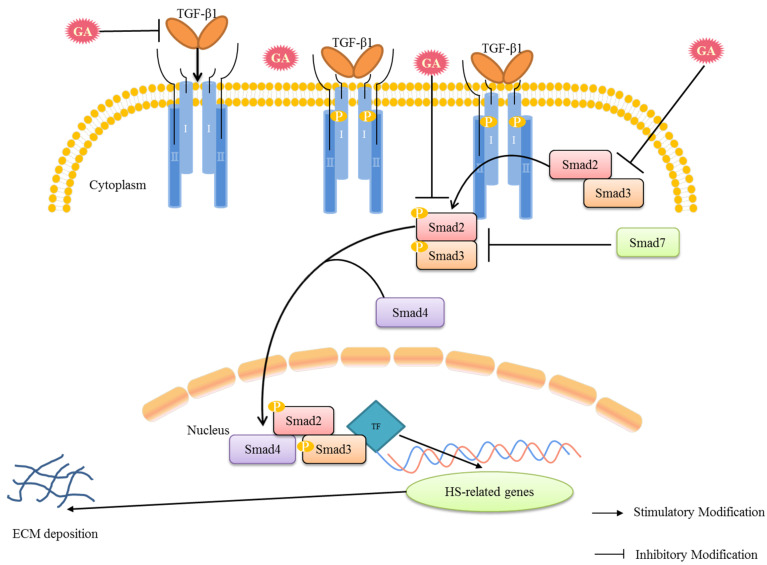
The HS potential inhibitory mechanism of GA via TGF-β/Smad signaling pathway. (I, type I receptors; II, type II receptors; GA, gallic acid; TF, the DNA transcription factor.)

TGF-β1 is crucial in promoting HS-related angiogenesis, myofibroblast differentiation, and matrix deposition [41]. The TGF-β1 signal transduction process involves binding the TGF-β1 ligand to the heteromeric complex of type II and type I receptors, followed by the phosphorylation of the type II receptor, which activates type I receptors. These type I receptors then activate downstream molecular signaling, thereby influencing the Smad2/3 signaling pathway (Figure 6) [42]. Smad (small mother against decapentaplegic) proteins are signal transduction and transcriptional regulators that mediate multiple signaling pathways [43]. There are eight different Smad proteins, which are mainly classified as comediator Smad (Co-Smad), receptor-regulated Smad (R-Smad), and inhibitory Smad (I-Smad) [20]. After being phosphorylated by type I receptors, Smad2 and Smad3 translocate to the nucleus with Smad4 (Co-Smad) to bind to DNA transcription factors. This regulates the abnormal deposition of ECM, which exacerbates HS [44,45]. Smad7 belongs to the I-Smad class and can compete with R-Smads and interact with type I receptors. This prevents the recruitment and phosphorylation of effector Smads and inhibits the TGF-β/Smad signaling pathway [9]. GA significantly suppressed TGF-β1-stimulated hypertrophic scar fibroblast (HSF) contraction in a dose- and time-dependent manner [37]. GA also induced growth inhibition, apoptosis, and necrosis in HSFs in a dose-dependent manner [16,46]. Moreover, GA had high antiproliferative activity and significantly increased the proportion of scar fibroblasts in the G0/G1 phase [47]. GA might potentially be developed as a treatment for patients with hypertrophic scars.

Although GA has been reported to regulate growth inhibition, apoptosis, and necrosis in HSF, its effects and mechanisms in rabbit ear HS models have not been investigated [46]. To investigate the role of GA in rabbit ear HS models, the therapeutic efficacy of GA was first evaluated through gross observation, histological evaluation, SEI, and VSS scores. The results showed that treatments with GA ointment significantly reduced HS. Subsequently, RNA sequencing technology was utilized to analyze the differential gene expression of normal rabbit ear tissues, rabbit ear tissues of the HS model, and rabbit ear tissues after GA treatments. The results demonstrated that the differentially expressed genes were primarily associated with the ECM signaling pathway, which is closely related to the formation of HS. TGF-β1, a mediator of ECM production and a stimulator of tissue regeneration and injury repair, is a critical factor in the pathogenesis of HS [48]. Reducing ECM during wound healing is one of the strategies to treat HS. Therefore, the roles of TGF-β1 and its downstream pathways in HS were explored by further RT-qPCR and Western blot experiments, and it was found that GA could improve HS by inhibiting the expression of TGF-β1, Smad2/3, and p-Smad2/3 proteins. Immunohistochemical staining for TGF-β1 in different groups was also performed in our study. As shown in Supplementary Figure S4, TGF-β1 could be suppressed after the GA treatment of HSs. Additionally, RT-qPCR analysis demonstrated that GA treatment reduced the expression of TRPC3, indicating that GA may also treat HS by downregulating TRPC3 expression.

CD31, expressed on vascular endothelial cells, is often used to measure angiogenesis [49,50]. HS formation is linked to angiogenesis [51]. Therefore, to further investigate the ability of GA to influence angiogenic processes during rabbit ear scar tissue formation, a quantitative comparison of the density of CD31-positive vasculature in scar tissue sections was performed using immunohistochemical staining. As shown in Supplementary Figure S3, the HSG5 group formed relatively fewer microvessels than the HS Group 2 weeks after the application of GA, and the same trend was also observed in the HSG1 and HSG2 groups. Moreover, prolonged wound healing usually results in hypertrophic scarring. In general, the epithelialized area was confirmed by macroscopic images and statistical analysis, and the time of complete epithelialization after wound modeling was related to wound healing [52]. As shown in Supplementary Figure S5, compared with the HS and HSM groups, the HSG and HSS groups significantly decreased the complete wound-epithelialization time, indicating that GA and silicone gel could accelerate wound healing while inhibiting scar formation.

This study provides evidence that GA exerts ameliorative effects on skin scar formation, potentially through modulation of the TGF-β1/Smad pathway (Figure 6). Nonetheless, HS is a complex condition involving multiple signaling cascades, with the TGF-β1/Smad and TRPC3 signaling pathways displaying crosstalk with other signaling pathways. Thus, it is crucial to investigate other possible mechanisms of action of GA on HS in forthcoming studies and delve deeper into its anti-fibrotic effects.

## 4. Materials and Methods

### 4.1. Materials

Different doses of GA ointment (composed of GA, honey, and black vinegar), matrix ointment (composed of honey and black vinegar), and silicone gel ointment (National Machinery Shanghai Trading Co., Ltd., Shanghai, China) were provided by the Department of Dermatology, Shuguang Hospital, affiliated with the Shanghai University of Traditional Chinese Medicine. Respiratory anesthetic isoflurane was purchased from Suzhou Kunchen Biotechnology Co., Ltd., Jiangsu, China. Sections of HE and Masson staining reagents were obtained from Wuhan Servicebio Biotechnology Co., Ltd., Hubei, China. The RNA primer sequences (synthesized by Shanghai Shanjin Biotechnology Co., Ltd., Shanghai, China) are shown in Supplementary Table S1. GAPDH (30201ES20) was purchased from Yeasen Biotechnology Shanghai Co., Ltd., Shanghai, China. TGF-β1 (MA1-34093) was purchased from ThermoFisher, Waltham, MA, USA. Smad2/3 antibody (ab202445) was purchased from Abcam, Cambridge, UK, and the p-Smad2/3 (AP1343) was obtained from ABclonal Technology Co., Ltd., Hubei, China. Secondary antibodies (7074S and A0216) were obtained from Cell Signaling Technology, Danvers, MA, USA and Beyotime Biotechnology, Shanghai, China.

### 4.2. Animals

Fifty New Zealand white male rabbits, 2.5–3.0 kg, were purchased from Chedun Experimental Animal Breeding Farm Ltd., in Songjiang District, Shanghai, and were acclimatized for one week before modeling. The animals were housed individually under standard conditions, and the experimental protocol was approved by the Ethics Committee of Shanghai University of Traditional Chinese Medicine (No. PZSHUTCM211018023).

### 4.3. Preparation of Rabbit Ear HS Models

The rabbits were fixed with a rabbit fixator and anesthetized by breathing. A perforator (KAI Medical) was used to create dermal wounds on the ventral side of the rabbit’s ear with a diameter of 8 mm and three rows of four wounds each for 12 wounds per ear [9]. The perforator must cut through the entire skin layer, peel it off without preserving the cartilage membrane, and press to stop the bleeding. Visible blood vessels should be avoided during the operation. After the wound was exposed and pressure was applied to stop bleeding, a local iodophor (Jiangxi Caoshanhu Disinfection Products Co., Ltd., Nanchang, China) was promptly given disinfection. Free access to food and water was provided, and the wound was cleaned continuously for 21 days [53]. The scar was entirely epithelialized when the wound healed independently, and the rabbit ear HS model was established. Any wounds with infection, necrosis, and unseen scarring were excluded from the study. The flowchart of the experiment schedule is shown in the Supplementary Figure S6.

### 4.4. Experimental Design and Treatment

The experiment was divided into seven groups: the model group, the matrix group, the low-dose GA ointment group (2.23%), the medium-dose GA ointment group (4.46%), the high-dose GA ointment group (11.15%), the silicone gel group, and the skin group (normal skin group, no treatment was performed throughout the experimental cycle). After epithelialization, a pharmacological intervention will be given at 0.1 mL (0.4*0.4*3.14*0.2) of ointment per wound. Meanwhile, weekly scar scoring was performed, and photographs were taken to record the development process of scars (based on the Vancouver Scar Scale rating form before drug treatment, weekly photographs of the scar site were taken, and the dressing was changed daily). For each experimental group, samples were collected one, two, and four weeks after treatment. One part was sent for pathological sectioning, and one was stored at −80 °C for protein and gene expression detection.

### 4.5. Histological Examination

Scar tissue was removed, fixed in 4% paraformaldehyde, and embedded in paraffin. Subsequently, sections were stained with hematoxylin and eosin (HE), and Masson’s trichrome stain was dehydrated, sealed, and observed under a light microscope (OLYMPUS BX41).

### 4.6. Scar Elevation Index and Vancouver Scar Scale Score Evaluation

The scar elevation index (SEI) was utilized to determine the degree of scar proliferation. SEI is defined as the height of the scar in relation to the normal skin perpendicular to the ear surface, measured from the top point of the scar/skin epithelium to the surface of the ear cartilage [54]. Measurements were made using Image-Pro Plus 6.0 software, and the SEI was measured for each wound. The Vancouver Scar Scale (VSS) scores were determined by three experimentalists simultaneously, and the scores were mean values.

### 4.7. RNA Sequencing Analysis

Samples of normal rabbit ear tissue (Skin group), rabbit ear of HS model (HS group), and rabbit ear tissue of GA treatment for HS (HSG2 group) were assayed for RNA quality and purity by Shanghai Personal Biotechnology Co., Shanghai, China, for transcriptome sequencing after passing the quality and purity of RNA. The primer sequence information is shown in Supplementary Table S1. The cDNA first and second strands were synthesized with six-base random primers and reverse transcriptase using purified mRNA from total RNA as a template. After the cDNA was converted to double-stranded DNA purified to construct the library, it was quantified using the Agilent Bioanalyzer 2100 system for high-sensitivity DNA analysis. After library construction, paired-end sequencing was performed on the libraries based on the Illumina HiSeq sequencing platform. Differentially expressed genes were identified by an absolute foldchange >1.2 or <0.83. KEGG pathways were enriched for differentially expressed genes with *p*-values less than 0.05, using ClusterProfiler (3.4.4) software.

### 4.8. Real-Time Quantitative Polymerase Chain Reaction (RT-qPCR)

The samples were lysed by adding a lysis solution (Yeasen Biotechnology Shanghai Co., Ltd.) to the tissue grinder. Total RNA was extracted based on the instructions, and the reverse transcription reaction was performed at 42 °C for 15 min and 95 °C for 3 min (Tiangen Biochemical Technology Co., Beijing, China). The samples were quantified by an ABI 7500 Fast real-time fluorescence quantitative PCR instrument based on 40 cycles of initial denaturation at 95 °C for 5 min and then 10 s at 95 °C and 30 s at 60 °C, all standardized with internal reference β-actin, and each template was repeated four times under the same conditions.

### 4.9. Western Blot

The scar tissue was homogenized with lysate for Western blot detection. Each protein sample was added to 20 times the volume of RIPA (Beyotime Biotechnology) lysate containing 1× phosphatase inhibitor (Beyotime Biotechnology) and 1× protease inhibitor (Beyotime Biotechnology), fully lysed, and centrifuged at 13,200 rpm for 5 min, and the supernatant was taken. Each component solution was added to the wells of a 96-well plate and placed at 37 °C for 30 min, the OD value was measured at 562 nm, and the protein concentration of each sample was determined based on the standard curve. The samples were separated by 8% SDS gel (Yeasen Biotechnology Shanghai Co., Ltd.), passed onto PVDF membrane, milk powder closed and recovered, washed, and the membranes were incubated with primary antibodies (GAPDH, TGF-β1, Smad2/3, and p-Smad2/3) overnight at 4 °C. Secondary antibodies were dissolved in a secondary antibody diluent based on the manufacturer’s instructions, incubated for two hours, washed with TBST, and developed in a Bio-Rad imager. GAPDH protein was used as an internal reference control, and then the protein bands were analyzed by Image J software. The detected protein grayscale values were normalized to determine the protein expression changes.

### 4.10. Statistical Analysis

All reported values were presented as mean ± SD. Data were analyzed using one-way analysis of variance (ANOVA) by GraphPad Prism 7 software, and the data were subsequently analyzed using Tukey’s post hoc multiple comparisons test. A *p* < 0.05 was considered statistically significant.

## 5. Conclusions

This study provided evidence that GA effectively improved the morphology and histological structure of HS tissues in rabbit ear models, potentially through the downregulation of the TGF-β/Smad and TRPC3 signaling pathways, thereby reducing ECM deposition and eliciting anti-scarring effects. The findings indicate that GA holds potential as an effective therapeutic agent for HS.

## Data Availability

Data is contained within the article and Supplementary Materials.

## Supplementary Materials

The following supporting information can be downloaded at https://www.mdpi.com/article/10.3390/ph16111514/s1. Supplementary Figure S1. Histological staining of all groups after the treatments. (HS: Model group, HSM: Matrix group, HSG1: Low-dose GA ointment group, HSG2: Medium-dose GA ointment group, HSG5: High-dose GA ointment group, HSS: Silicone gel group, Skin: Normal skin group). Supplementary Figure S2. Gene expression between the skin, HS and HSG2 groups. Supplementary Figure S3. Immunohistochemical staining for CD31+ blood vessels in different groups. Black arrow: microvascular vessel; Magnification = 200; Bar = 100 μm. Significant difference between HS group and other groups (* *p* < 0.05, ** *p* < 0.01, n = 3); Significant difference between HSM group and other groups (# *p* < 0.05, ## *p* < 0.01, n = 3). Data was analyzed using one-way ANOVA with post-hoc Tukey’s multiple comparisons test. (HS: Model group, HSM: Matrix group, HSG1: Low-dose GA ointment group, HSG2: Medium-dose GA ointment group, HSG5: High-dose GA ointment group, HSS: Silicone gel group, Skin: Normal skin group.) Supplementary Figure S4. Immunohistochemical staining for TGF-β1 in different groups. Significant difference between HS group and other groups (* *p* < 0.05, ** *p* < 0.01, n = 3); Significant difference between HSM group and other groups (# *p* < 0.05, ## *p* < 0.01, n = 3). Data was analyzed using one-way ANOVA with post-hoc Tukey’s multiple comparisons test. (HS: Model group, HSM: Matrix group, HSG1: Low-dose GA ointment group, HSG2: Medium-dose GA ointment group, HSG5: High-dose GA ointment group, HSS: Silicone gel group, Skin: Normal skin group.) Supplementary Figure S5. HSG significantly decreased complete wound-epithelialization time. Significant difference between HS group and other groups (* *p* < 0.05, ** *p* < 0.01, n = 9); Significant difference between HSM group and other groups (# *p* < 0.05, ## *p* < 0.01, n = 9). Data was analyzed using one-way analysis of variance ANOVA with post-hoc Tukey’s multiple comparisons test. (HS: Model group, HSM: Matrix group, HSG1: Low-dose GA ointment group, HSG2: Medium-dose GA ointment group, HSG5: High-dose GA ointment group, HSS: Silicone gel group, Skin: Normal skin group.) Supplementary Figure S6. The experimental schedule. Supplementary Table S1. Primer sequence information.
