# Supplementary material for: Gallic Acid Treats Hypertrophic Scar in Rabbit Ears via the TGF-β/Smad and TRPC3 Signaling Pathways
Source: Pharmaceuticals (Basel). 2023 Oct 24;16(11):1514. doi: 10.3390/ph16111514 (PMC10675562; doi:10.3390/ph16111514)
Supplement: Supplementary file 1 [file pharmaceuticals-16-01514-s001.zip › pharmaceuticals-2567349-supplementary.pdf]

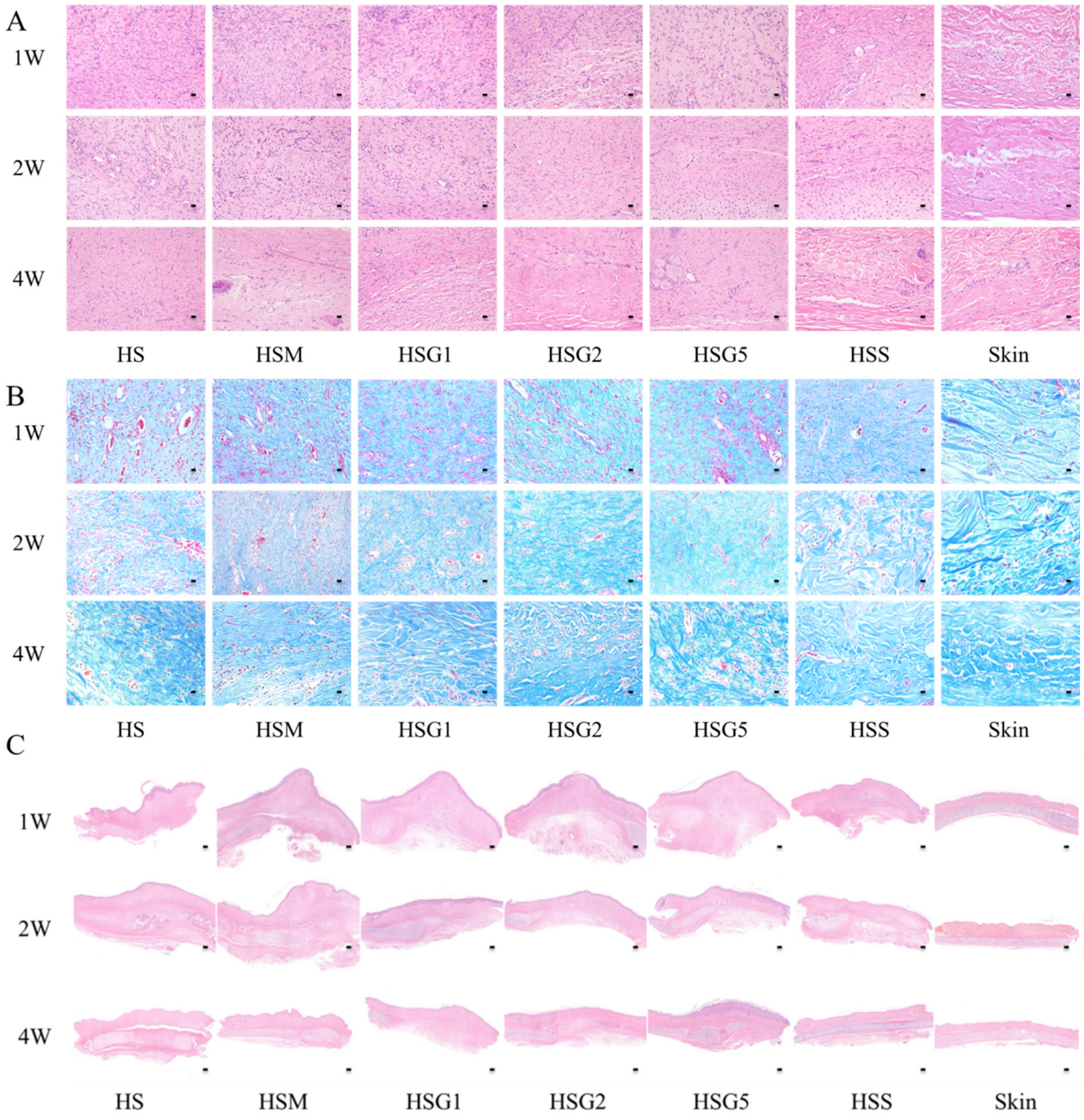

Supplementary Figure S1. Histological staining of all groups after the treatments. (HS: Model group, HSM: Matrix group, HSG1: Low-dose GA ointment group, HSG2: Medium-dose GA ointment group, HSG5: High-dose GA ointment group, HSS: Silicone gel group, Skin: Normal skin group)

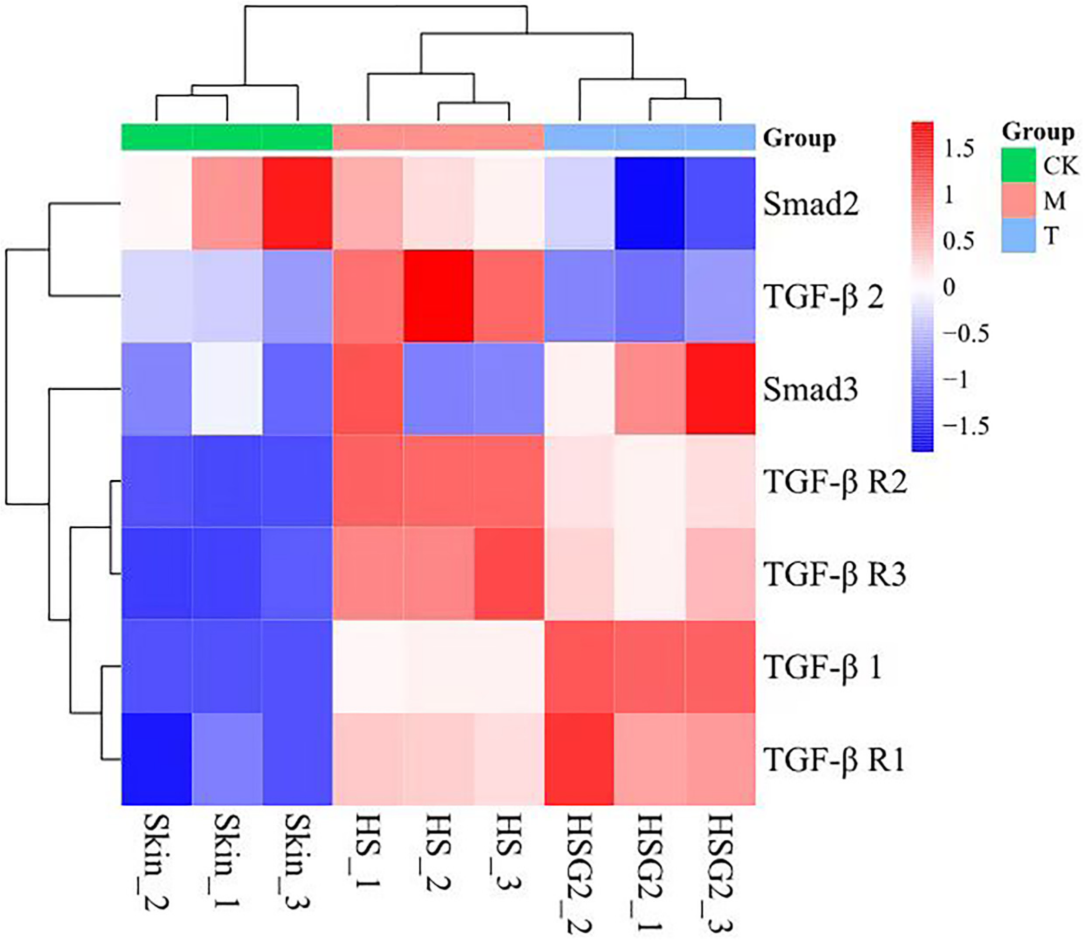

Supplementary Figure S2. Gene expression between the skin，HS and HSG2 groups.

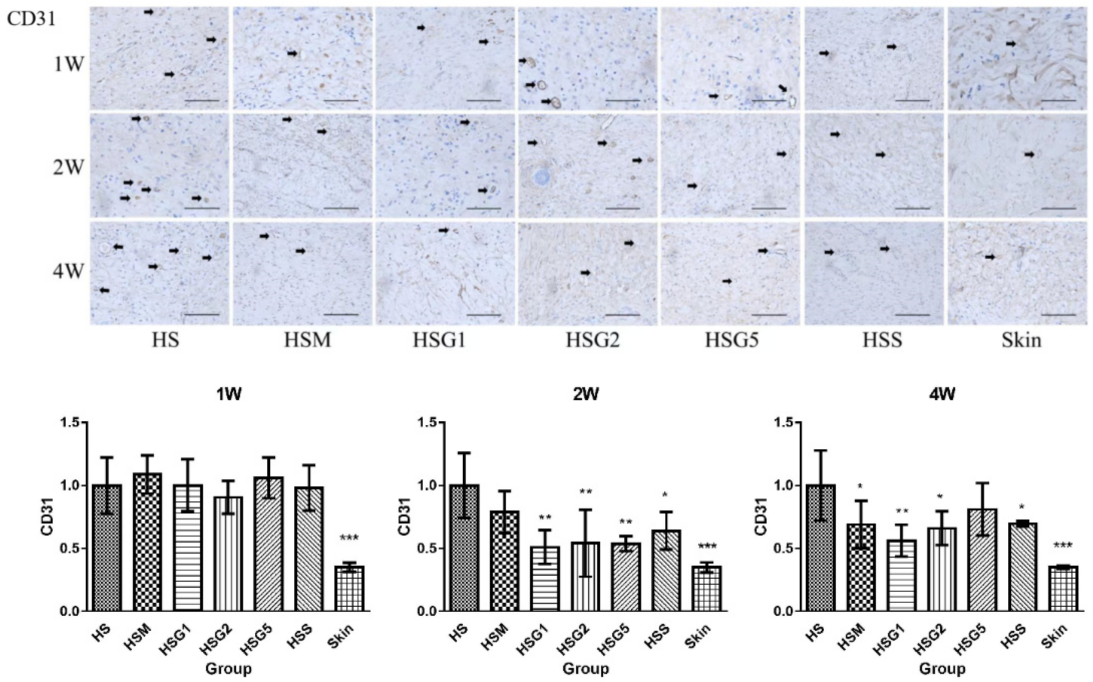

Supplementary Figure S3. Immunohistochemical staining for CD31+ blood vessels in different groups. Black arrow: microvascular vessel; Magnification = 200; Bar =100 μm. Significant difference between HS group and other groups (*p < 0.05, **p < 0.01, n = 3); Significant difference between HSM group and other groups (#p < 0.05, ##p < 0.01, n = 3). Data was analyzed using one-way ANOVA with post-hoc Tukey's multiple comparisons test. (HS: Model group, HSM: Matrix group, HSG1: Low-dose GA ointment group, HSG2: Medium-dose GA ointment group, HSG5: High-dose GA ointment group, HSS: Silicone gel group, Skin: Normal skin group.)

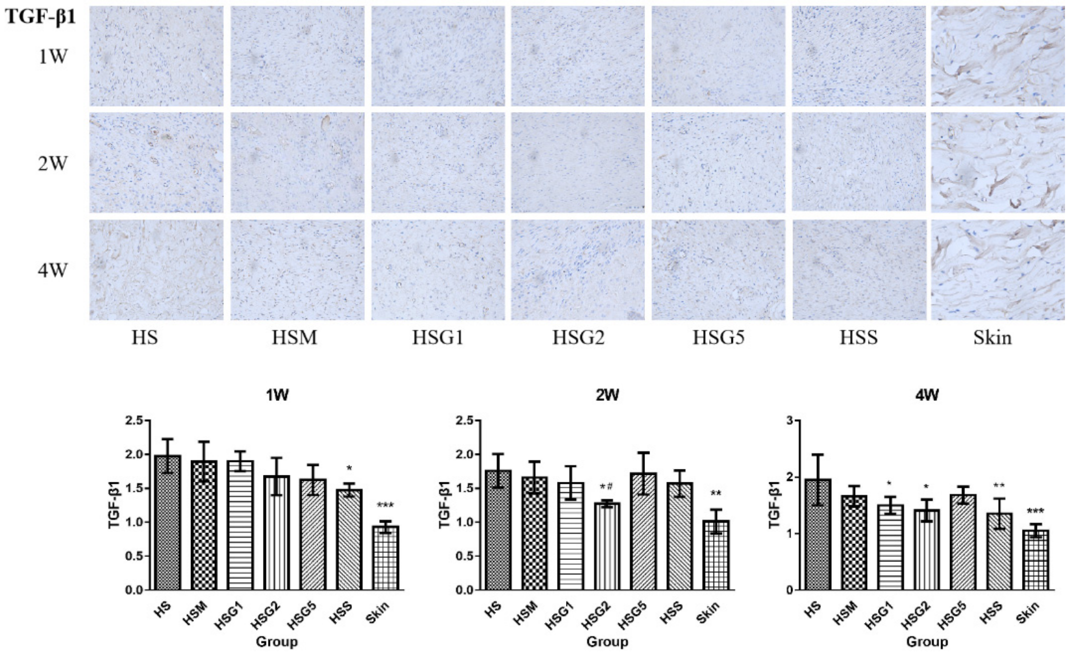

Supplementary Figure S4. Immunohistochemical staining for TGF-β1 in different groups. Significant difference between HS group and other groups (*p < 0.05, **p < 0.01, n = 3); Significant difference between HSM group and other groups (#p < 0.05, ##p < 0.01, n = 3). Data was analyzed using one-way ANOVA with post-hoc Tukey's multiple comparisons test. (HS: Model group, HSM: Matrix group, HSG1: Low-dose GA ointment group, HSG2: Medium-dose GA ointment group, HSG5: High-dose GA ointment group, HSS: Silicone gel group, Skin: Normal skin group.)

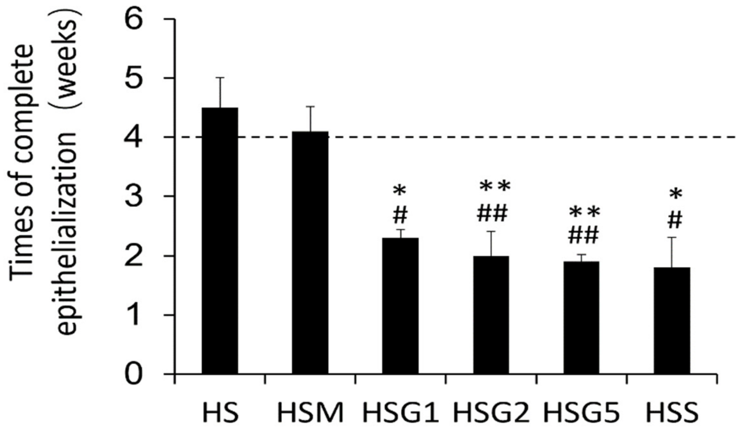

Supplementary Figure S5. HSG significantly decreased complete wound-epithelialization time. Significant difference between HS group and other groups (*p < 0.05, **p < 0.01, n = 9); Significant difference between HSM group and other groups (#p < 0.05, ##p < 0.01, n = 9). Data was analyzed using one-way analysis of variance ANOVA with post-hoc Tukey's multiple comparisons test. (HS: Model group, HSM: Matrix group, HSG1: Low-dose GA ointment group, HSG2: Medium-dose GA ointment group, HSG5: High-dose GA ointment group, HSS: Silicone gel group, Skin: Normal skin group.)

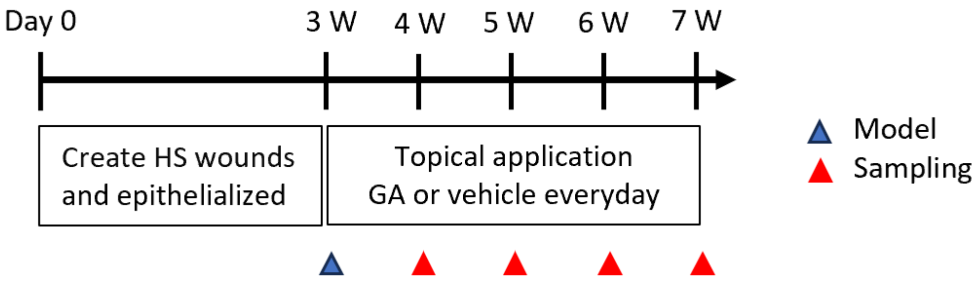

Supplementary Figure S6. The experimental schedule

Supplementary Table 1. Primer sequence information.

Gene	Sequence（5`-3`）	Annealing Temperature, °C	Product Size, bp
TGF-β1	Forward: CCCAAGTGATGATGAGGTGC<br>Reverse: CCTTGCCAAAGAAGCCTGAG	60	134
Smad2	Forward: TACTGTCCAATGTGAACCGAAAC<br>Reverse: AGGGTGCCAGCCGTATCTC	60	167
Smad3	Forward: CATTCCATCCCCGAGAACAC<br>Reverse: GCTGTGGTTCATCTGGTGGTC	60	126
TRPC3	Forward: TTCATTGAGTCGTGTCAAACTTG<br>Reverse: ACACTTGATGGCCACGGTC	60	139
β-actin	Forward: TGACCAACTGGGACGACATG<br>Reverse: GTGTTGAACGTCTCGAACATGA	60	160
